# The pH Dependence of Polymerization and Bundling by the Essential Bacterial Cytoskeltal Protein FtsZ

**DOI:** 10.1371/journal.pone.0019369

**Published:** 2011-06-28

**Authors:** Raúl Pacheco-Gómez, David I. Roper, Timothy R. Dafforn, Alison Rodger

**Affiliations:** 1 Molecular Organization and Assembly in Cells Doctoral Training Centre, University of Warwick, Coventry, United Kingdom; 2 School of Life Sciences, University of Warwick, Coventry, United Kingdom; 3 Department of Chemistry, University of Warwick, Coventry, United Kingdom; 4 Department of Biosciences, University of Birmingham, Edgebaston, Birmingham, United Kingdom; University of Pennsylvania, United States of America

## Abstract

There is a growing body of evidence that bacterial cell division is an intricate coordinated process of comparable complexity to that seen in eukaryotic cells. The dynamic assembly of *Escherichia coli* FtsZ in the presence of GTP is fundamental to its activity. FtsZ polymerization is a very attractive target for novel antibiotics given its fundamental and universal function. In this study our aim was to understand further the GTP-dependent FtsZ polymerization mechanism and our main focus is on the pH dependence of its behaviour. A key feature of this work is the use of linear dichroism (*LD*) to follow the polymerization of FtsZ monomers into polymeric structures. *LD* is the differential absorption of light polarized parallel and perpendicular to an orientation direction (in this case that provided by shear flow). It thus readily distinguishes between FtsZ polymers and monomers. It also distinguishes FtsZ polymers and less well-defined aggregates, which light scattering methodologies do not. The polymerization of FtsZ over a range of pHs was studied by right-angled light scattering to probe mass of FtsZ structures, *LD* to probe real-time formation of linear polymeric fibres, a specially developed phosphate release assay to relate guanosine triphosphate (GTP) hydrolysis to polymer formation, and electron microscopy (EM) imaging of reaction products as a function of time and pH. We have found that lowering the pH from neutral to 6.5 does not change the nature of the FtsZ polymers in solution—it simply facilitates the polymerization so the fibres present are longer and more abundant. Conversely, lowering the pH to 6.0 has much the same effect as introducing divalent cations or the FtsZ-associated protein YgfE (a putative ZapA orthologue in *E. coli*)—it stablizes associations of protofilaments.

## Introduction

**Figure 1:**
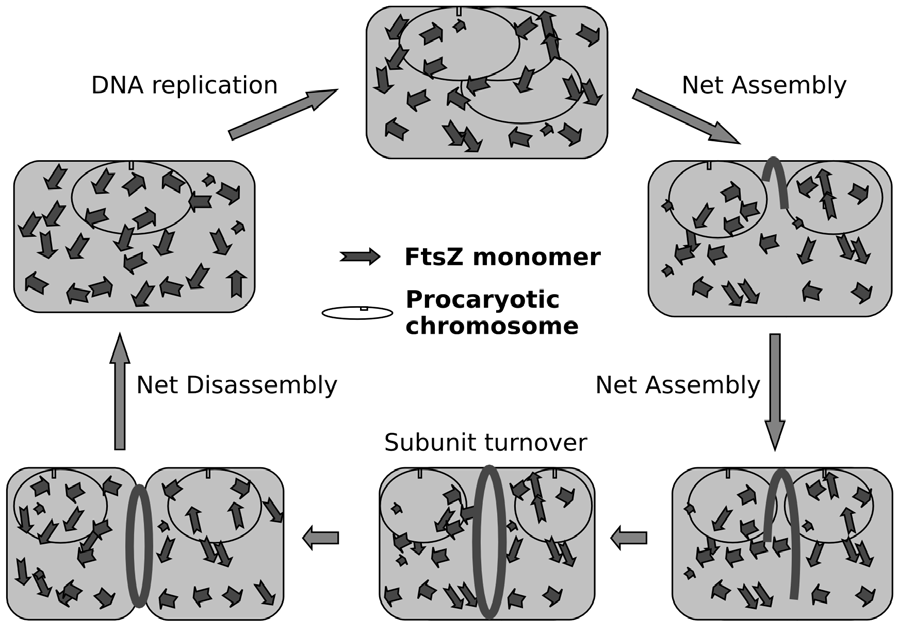
Simplified schematic illustration of the dynamic behaviour of FtsZ during the cell division cycle. Cell growth occurs continuously and when the cell reaches an appropriate size, the plasma membrane and cell wall grow inwards to divide the cell in two between the two sets of daughter chromosomes.

There is a growing body of evidence that bacterial cell division is an intricate coordinated process of comparable complexity to that seen in eukaryotic cells [1]. Bacterial cytokinesis is achieved by a coordinated set of events governed by the divisome, at the heart of which lies the bacterial tubulin homologue, FtsZ [2]. In simple terms, the primary task of the bacterial cell is cell-duplication which proceeds, by binary fission, in two phases: (i) the single, circular DNA molecule replicates, and (ii) division occurs by the invagination of the plasma membrane and the laying down of new cell wall material between the two chromosomes to produce two separate daughter cells. The key molecule for the second phase is FtsZ which, with the aid of a number of other proteins, small molecules and ions, assembles and binds to the correct part of the cell membrane then pulls it inwards as illustrated in the oversimplified schematic of Figure 1.

The dynamic assembly of FtsZ is fundamental to its activity. *Escherichia coli* FtsZ polymerizes in the presence of GTP into protofilaments (a single stranded longitudinal head-to-tail assembly of FtsZ monomers) and adopts protofilament-derived high order structures under many conditions [3], [4]. The *in vivo* arrangement of FtsZ is not yet known, but it is thought to contain 6 to 7 protofilaments [5]. FtsZ polymerization is a very attractive target for novel antibiotics given its fundamental and universal function [6]. In this study our aim was to understand further the GTP-dependent FtsZ polymerization mechanism. The mechanisms of protofilament formation and their bundling, together with the role of GTPase activity on FtsZ polymer structure and dynamics, are complex and many aspects remain to be elucidated, as illustrated by the recent careful work by Chen and Erickson [7]. The work reported in this paper is part of a larger project to understand how FtsZ and its associated proteins control bacterial cytokinesis. In this study of FtsZ, our focus is on the pH dependence of its behaviour.

As in our previous work on the effects on FtsZ of Ca^2+^ and the cell division protein YgfE [8], [9], a key feature of this work is the use of linear dichroism (*LD*) to follow the polymerization of FtsZ monomers into polymeric structures. *LD* is the differential absorption of light polarized parallel and perpendicular to an orientation direction (in this case that provided by shear flow)—the signal is zero in the absence of an oriented sample and non-zero when an absorbing sample is oriented. It thus readily distinguishes between FtsZ polymers and monomers. It also distinguishes FtsZ polymers and less well-defined aggregates, which light scattering methodologies do not. The other key tool required for this work was an accurate phosphate release assay since a key factor in the control of FtsZ structure is the GTP-dependent assembly of FtsZ into protofilaments followed by the hydrolysis of GTP to GDP by an active site formed between two associated FtsZ monomers. The GDP-containing polymers are thought to preferentially adopt a curved structure, and so place the overall polymer under stress. GDP to GTP exchange is also believed to occur along the full length of the FtsZ polymer [1].

Whilst the optimal environmental pH for *E. coli* bacteria is around neutral, they can grow and divide within a much greater range than this [10]. Mukherjee and Lutkenhaus [11] studied different conditions that affect the kinetics of polymerization and depolymerization of FtsZ and found that FtsZ readily formed polymers at pH = 6.0, 6.5 and 7.2. They concluded that the only difference between the conditions was the abundance of the polymers (more abundant at pH 6.5). Sheffers in the context of a study on the effect of MinC on FtsZ polymerization noted that FtsZ polymers form more quickly and last longer at pH 6.5 than at pH 7.5, though the implication of their data is that the maximum abundance at the two pH's is the same within experimental error [12]. Mendieta et al. [13] while studying the sodium and potassium dependence of FtsZ polymerization, also noted pH dependence of FtsZ GTPase activity (again using malachite green) and light scattering. They ascribed the pH dependence completely to a proposed protonation of the GTP without any recognition of the effect of FtsZ fibre bundling that also occurs at lower pH. However, we know from previous work that the effect of bundling when induced by Ca^2+^ or YgfE [8], [9] is to slow down GTPase activity and to reorient the GTP indicating that the pH-induced bundling may be sufficient to reduce GTPase activity calling the conclusions of reference [13] into question.

In any case, the conclusion from the literature is that the effect of pH on FtsZ polymerization is not quite clear. In this work we have therefore undertaken a careful study of the polymerization of FtsZ over a pH range from 6.0 to 7.0 using right-angled light scattering to probe mass of FtsZ structures, *LD* to probe real-time formation of linear polymeric fibres and GTP reorientation, a specially developed phosphate release assay to relate guanosine triphosphate (GTP) hydrolysis to polymer formation, and electron microscopy (EM) imaging of reaction products as a function of time and pH.

## Materials and Methods

### Chemicals and reagents

All chemicals used were of analytical grade and only molecular biology grade water (0.1 µm filtered) or 18.2 MΩ cm water was used. Unless otherwise stated, the providers were: Sigma-Aldrich (Poole, Dorset, UK), Calbiochem (USA), Fisher Scientific (UK), Fluka Chemika (Germany) or Helena Biosciences (UK).

All assays were carried out in MES (2-[N-Morpholino]ethane sulfonic acid) buffer solutions which were prepared and then adjusted to the required pH. The final pHs used in this work were 6.0, 6.5, and 7.0 unless otherwise stated.

### FtsZ

The key to the success of this work was the production of highly monomeric and correctly folded *E. coli* FtsZ (FtsZ), which is mono-disperse, as assessed by analytical ultracentrifugation and of 99% or greater purity as assessed by SDS-PAGE gel electrophoresis. It is our experience that whilst overexpression of *E. coli* FtsZ is routine and predictable, there is significant batch-to-batch variation in the quality of the final purified protein produced and in its ability to polymerise and hydrolyse GTP. The use of selective affinity tags such as those used for immobilised metal affinity chromatography, which can produce large quantities of protein, in our hands did not produce the required monomeric, correctly folded, active protein. Thus we developed production methodologies that gave FtsZ of the required standard for the biophysical studies and developed protocols to assess rigorously the functional quality of our protein preparations prior to analysis (see below for details). The methodology developed to purify *E. coli* FtsZ allowed us to obtain a highly pure protein, which consisted of 95% monomeric sample that could polymerize. The protein stock solutions were aliquoted in 100 µl fractions and stored at −80°C until the day of use. Purified *E. coli* FtsZ stored in this way is stable for at least 2 years with no apparent loss of GTPase or polymerisation activities. *E. coli* FtsZ has no tryptophan residues and very few tyrosine residues making absorbance at 280 nm an unsuitable measure of protein concentration. The protein concentration was therefore determined using the Bradford method colorimetric protein assay with BSA as a standard [14].

### Protein expression and purification

*E. coli* FtsZ was produced and purified largely following the method described by Mukherjee and Lutkenhaus [15]. However, some modifications were introduced: the pooled, dialysed ion-exchange DEAE-sepharose column fractions were subject to a 20% ammonium sulfate cut that removes the acetate kinase activity from the FtsZ preparation and the last chromatography step was omitted. Instead, the 20% ammonium sulfate cut fraction was further purified by ion-exchange chromatography on DEAE-sepharose. The sequence of steps in the scheme are detailed below.

#### Overproduction of E. coli FtsZ

To overproduce *E. coli* FtsZ, *Escherichia coli* competent cells B834(DE3) (Novagen) were transformed with the plasmid pETFtsZ in which the *E.coli* FtsZ gene was cloned into the NcoI and BamHi sites of pET15b (Novagen). A 10 ml liquid culture of Luria-Bertani (LB) containing ampicillin (final concentration 100 µg/mL) was inoculated with a fresh colony of the transformed cells and grown overnight with shaking at 37°C and 180 rpm in non-baffled 2 L flasks. The overnight culture was diluted into 3 L of fresh LB medium with ampicillin (100 µg/mL) and grown at 37°C in non-baffled 2 L flasks. At an OD_600_ (optical density at 600 nm) of 0.4, the culture was induced with isopropyl-β-D-thiogalactopyranoside (ITPG, final concentration 1 mM) and the growth continued for another 3 hours. In our hands induction at this point was critical, induction beyond an OD_600_ of 0.5 resulted in a much lower yield of FtsZ. The culture was then chilled and harvested at 4°C by centrifugation (10,000 g for 10 minutes) and washed once with cold 10 mM Tris-HCl, pH 7.9. From this step onwards, all procedures were carried out at 4°C.

#### Lysis of the bacterial cells and ammonium sulfate precipitation

The culture was then centrifuged (10,000 g for 10 minutesand the pellet was resuspended in 20 ml of Tris-HCl, (pH 7.9, 50 mM) with KCl (50 mM), ethylendiaminetetraacetic acid (EDTA, 1 mM) and glycerol (10%) (buffer A). It was then lysed by passage two times through a French press. The resulting extract was then centrifuged at 10,000 g for 10 minutes to remove unbroken cells and debris and the supernatant was centrifuged afterwards at 75,600 g for 90 min to remove the membrane fraction. The supernatant was then collected and 30% ammonium sulfate (16.6 g/100 mL of extract) was slowly added with stirring for 20 minutes. The ammonium sulfate precipitate was then recovered after centrifugation at 20,000 g for 10 minutes and resuspended extensively in buffer A. The resuspended pellet in buffer A was dialyzed overnight against buffer A with two changes of 1 L each.

#### Purification of FtsZ by ion-exchange chromatography using DEAE-Sepharose

A HiPrep 16/10 DEAE FF (GE Healthcare) column was used for anion exchange chromatography of FtsZ. In our experience, rigorous cleaning of the column between purification runs was required to ensure reproducibility between batches of protein. The dialyzed ammonium sulfate fraction was loaded onto a DEAE-sepharose column (bed volume, 20 mL) equilibrated with buffer A, washed with 5 bed volumes of buffer A and eluted with 200 mL of a 50 mM to 1 M KCl gradient at a flow rate of 0.2 mL/min. Fractions of 2 mL were collected and the peak fractions of FtsZ eluted at around 200–250 mM KCl. Proteins were monitored by the UV absorbance at 280 nm and protein elution was detected by the increase of the absorbance at 280 nm. Since FtsZ has no tryptophan residues and as a result has an unusually low absorbance at 280 nm, the purification of the protein was monitored by sodium dodecyl sulfate–polyacrylamide gel electrophoresis and the protein concentration of fractions is assayed with the Bio-Rad Assay Reagent.

#### Further purification

The fractions that appeared as a single band with little or no contamination in the SDS–PAGE were pooled together and dialysed extensively against buffer A (3 changes of 3 Ls each). A co-purifying acetate kinase activity has previously been reported in the pooled fractions [16]. To overcome this problem, the pooled dialysed fractions were subject to a 20% ammonium sulfate cut (slowly added with stirring) that removes the acetate kinase activity from the FtsZ preparation. The ammonium sulfate precipitate was then recovered after centrifugation at 20,000 g for 10 minutes and resuspended extensively in buffer A. The resuspended pellet was dialyzed overnight against buffer A with 3 changes of 3 Ls each.

At this stage FtsZ was more than 95% pure as visualized by SDS–PAGE analysis. FtsZ was further purified by DEAE-sepharose column (HiPrep 16/10 DEAE FF) following the steps outlined above. The fractions that showed a single band on SDS–PAGE were pooled together and dialysed extensively overnight in buffer A with 3 changes of 3 Ls each. The concentration was then assayed and the fractions aliquoted in 100 µl and stored at −80°C. Purified FtsZ stored in this way is stable for at least 2 years with no apparent loss of GTPase or polymerisation activities (as judged by 90° angle light scattering).

### Circular dichroism

Circular dichroism is the difference in absorbance of left and right circularly polarized light. With proteins the observed spectrum due to the backbone chromophores can be deconvoluted to give an estimate of the secondary structure of the protein. The *CD* spectrum of FtsZ was collected using a Jasco J-715 spectropolarimeter in a 0.01 mm pathlength cuvette.

### Kinetics studies of FtsZ polymerization

For the standard polymerization assays, FtsZ to a final concentration of 11 µM was incubated at 25°C in MES-KOH (50 mM), KCl (50 mM), and MgCl_2_ (10 mM) for 10 minutes. pH was varied between 6.0 and 7.0. GTP or GDP as required were then added and the effect measured.

### Right-angle light-scattering assay

Right-angle light-scattering measurements, based on the method developed by Mukherjee and Lutkenhaus [17], were used to follow the kinetics of the FtsZ assembly (assumed to be polymerization, though *LD* and EM are required to confirm this). High levels of light scattering were assumed to correlate with extensive polymerization; no change in light scattering to a steady state; and a decrease in light scattering to depolymerization. Experiments were undertaken at room temperature (∼25°C) using a Perkin Elmer LS50B spectrofluorimeter (which was calibrated daily using water and the auto-calibration routine) in a 0.3 cm path length fluorescence cuvette. The excitation and emission wavelengths were set at 450 nm and the excitation and emission slit widths were set at 2.5 nm. A ‘fluorescence’ (actually light scattering not true fluorescence) baseline was collected for approximately 10 minutes. The polymerization reaction was initiated by the addition and thorough mixing (with a micropipette) of GTP (final concentration 0.2 mM) freshly thawed on the day of use. The reading at time zero is the first reading following nucleotide addition (about 10 s after addition of GTP). The net change in light scattering following nucleotide addition was plotted as a function of time.

### Linear dichroism (LD)

*LD* measurements were performed at room temperature (∼25°C) using a Jasco J-715 spectropolarimeter adapted for *LD* spectroscopy. No *LD* signals are expected from monomeric units of FtsZ (approximately spheres) since they will not align individually, whereas polymeric forms of FtsZ will align and give an *LD* signal. Samples were aligned in the light beam using a custom made Couette cell (Crystal Precision Optics, Rugby, now available from Dioptica Scientific Ltd. *via* Kromatek Ltd., Great Dunmow, UK) which consists of a cylindrical cross section sleeve with a removable quartz capillary (sealed at one end with Araldite Rapid®) held centrally with respect to its circular face with a quartz rod inserted into the capillary. The annular gap between a quartz rod suspended from the demountable lid and the inner capillary wall was 0.25 mm. The rod and the centre of the capillary were aligned so that the capillary was able to rotate freely inducing a shear force across the sample and laminar flow. Extensive validation of this cell has been undertaken [18], [19]. Data were collected using an interval scan measurement program that was available within the Jasco software. This enabled single full wavelength scans from 350–210 nm to be collected every minute at a scanning speed of 200 nm min^−1^, data pitch 0.5 seconds. Thus polymerization kinetics was monitored across the whole wavelength range of the spectrum. Baselines of data collected after depolymerisation of FtsZ were substracted from all spectra. 210 nm was the lowest wavelength where valid data could be collected at 11 µM FtsZ. The time taken to load and assemble the capillary *LD* unit and start the analysis (dead time) was about 60 seconds. In some instances, the rod was carefully removed to add another reagent at a specified time point.

#### Correction method for background light scattering in LD

The correction method used for light scattering is based on the correction for the background turbidity of brain microtubules described by J. Nordh *et al.* [20] and modified by R. Marrington [21]. It is based on the equation *LD*_scattering_(λ) = *a*λ^−*k*^. Where *LD*(λ) is the *LD* signal at wavelength λ. If λ is chosen to be outside the absorbance region of the sample, then the measured *LD* is only due to scattering. *a* is a constant and *k* is the exponent which varies between preparations taking values between the values 2.0–3.5. *a* and *k* are determined to ensure the measured *LD* and the scattering correction overlay outside the absorbance region.

### Spectrophotometric assay for the quantification of inorganic phosphate

The inorganic phosphate (PO_4_^3−^_,_ P_i_) was quantified using a method derived from an assay originally described by Webb [22] where PNP (purine nucleoside phosphorylase) converts MESG (2-amino-6-mercapto-7-methylpurine riboside) to ribose 1-phosphate and 2-amino-6-mercapto-7-methlpurine in the presence of inorganic phosphate (P_i_) with an associated change in the absorbance spectrum. For our purposes, the change in absorption at 360 nm allowed quantification of the inorganic phosphate consumed in the reaction at the pHs studied.

The assay proceeded by mixing the non-phosphate producing components in a cuvette, incubating the solution for 10 minutes at room temperature, zeroing the instrument at 360 nm and monitoring the absorbance during this time to check for any P_i_ contamination. P_i_ (for calibration) or GTP was then added. The change in absorbance at 360 nm was plotted as a function of the P_i_ concentration for the calibration curves. The GTP experiments were expressed in terms of a percentage of the theoretical maximum determined from the calibration curves.

### Electron Microscopy (EM)

The fibres of FtsZ formed at different time points under the different conditions described in this work were visualized using negative staining by transmission electron microscopy. Samples for electron microscopy (EM) were prepared by incubating FtsZ (11 µM) for 10 min at 25°C in the polymerisation buffer (50 mM MES-KOH, pH 6.5, 50 mM KCl, and 10 mM MgCl_2_) after which time GTP (final concentration 0.2 mM) was added. 5 µl of the sample mixture were then withdrawn at different time points for EM. Carbon-coated 400 mesh Cu carbon grids were glow-discharged prior to negative staining by placing them carbon side up (noted in the box) onto a glass slide and inserting them in the glow discharge instrument for 20 seconds at 10 mA (a pale purple glow). Samples were stained using a filtered (0. 2 µm filter) uranyl acetate stock solution (1%, UA). The negative staining was done by placing the sample (5 µl) onto the carbon side of the grid for 1 minute then washing the sample with three drops of the 1% UA solution. After 45 s the surface was blotted from the sample by gently touching/dragging the grid parallel or perpendicular to the filter paper. Grids were stored in a desiccator or a cool dry place, preferably in the dark and then viewed using a JOEL (Tokyo, Japan) transmission electron microscope model 1200EX at a magnification of ×20,000, ×75,000, and/or ×150,000.

## Results

**Figure 2:**
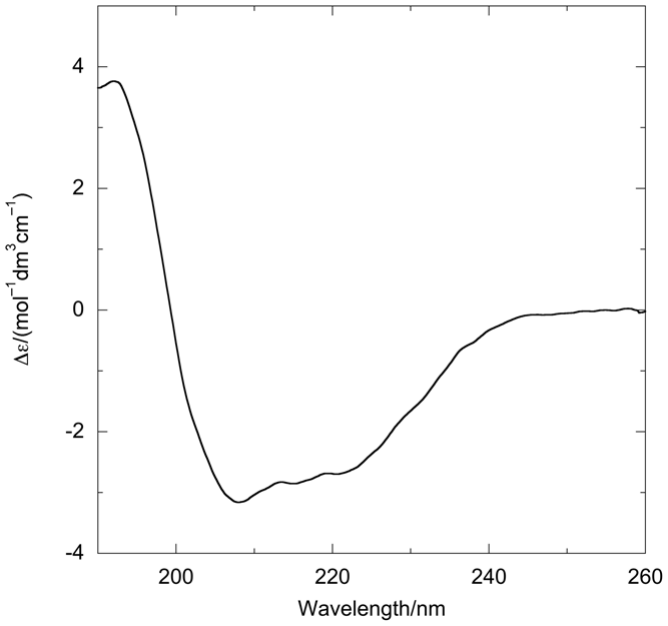
*CD* spectrum of FtsZ (22 µM) from the average of 3 independent data collections in Tris-HCl (50 mM, pH 7.9), KCl (50 mM), EDTA (1 mM) and glycerol (10%). *CD* spectra were measured using a 0.012 mm demountable cuvette (determined by measuring the absorbance of potassium chromate) at room temperature and data converted to Δε using amino acid concentration.

The *CD* spectrum of FtsZ in terms of a residue concentration (40.3 kDa, 382 residues) of Figure 2 indicates the secondary structure of FtsZ was correctly folded. The secondary structure fitting using CDsstr [23], [24] suggests FtsZ in solution is 21% α-helix; 7% polyproline II helix; 7% 3–10 helix; 14% β-sheet; 16% beta turn and 35% ‘other’ which accords well with the PDB entry 1FSZ for *Methanococcus jannaschii* FtsZ which has 38% helix; 27% β-sheet and turn; and 35% ‘other’.

FtsZ has been previously reported to polymerize over a broad pH range [11], [17]. We have previously examined the effect of the pH on the formation of FtsZ polymers by light scattering and electron microscopy [17]. In this work the effect of adding GTP to FtsZ was followed using a number of techniques as detailed below to provide complementary views of the process.

### pH as a function of time

The pH throughout the polymerization reactions remained constant within experimental error, thus any differences in behaviour of FtsZ and GTP as a function of pH can be attributed to the pH effect on the GTP-dependent polymerisation of FtsZ and not to a change in the pH after GTP addition.

### Right-angled light-scattering of FtsZ/GTP systems

**Figure 3:**
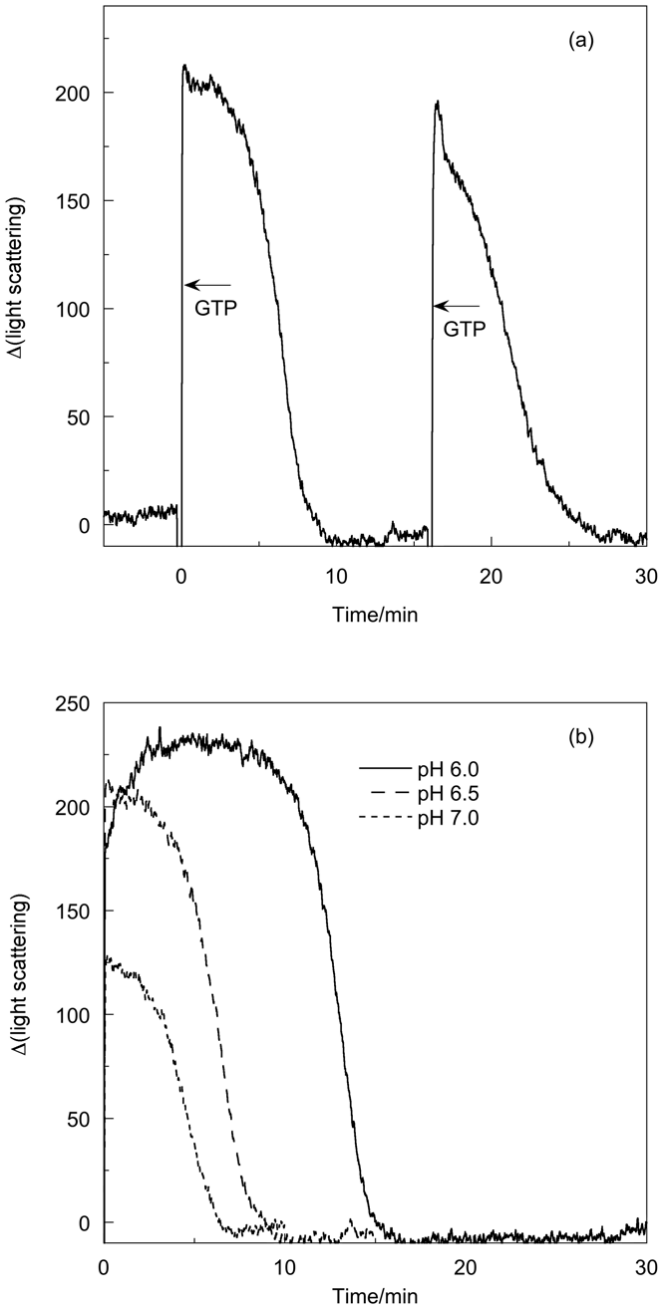
Light scattering of FtsZ as a function of pH. FtsZ (11 µM) in (a) MES buffer (50 mM pH 6.5), KCl (50 mM) and MgCl_2_ (10 mM) with addition GTP (0.2 mM) at *t* = 0 and at *t* = 16 min, and (b) in MES buffer (50 mM pH as indicated on the figure), KCl (50 mM) and MgCl_2_ (10 mM) with addition of GTP (0.2 mM) at *t* = 0. Data were collected every second at room temperature in a 0.3 cm path length fluorimeter cuvette with excitation and emission wavelengths set at 450 nm.

The effect on the right-angled light-scattering signal of the addition of excess GTP to FtsZ in polymerization buffer is shown in Figure 3a. After an initial sharp increase, there is a steady state period (∼2–4 min), followed by a decrease back to baseline all within 10 min. Further additions of GTP repeat the cycle, though addition of GDP and GMP (data not shown) have no such effect. These results are in accord with FtsZ undergoing rapid polymerisation and reaching a steady state under the conditions used for these experiments, where excess GTP is initially added. The plateau indicates a state of constant polymer mass after which there is a rapid decrease in light scattering, which indicates the FtsZ is depolymerizing.

Figure 3b shows the GTP-dependent polymerisation of FtsZ at pH 6.0, pH 6.5 and pH 7.0. The pH 6.0 sample shows greatest light scattering and takes longest time to reach its plateau region. The plateau region is also longest at pH 6.0 and shortest at pH 7.0. As discussed below, this is related to the rate of GTP turn-over. Depolymerisation occurs slightly more quickly (∼4 mins) at pH 7 than at pH 6 (∼6 mins).

### Linear dichroism of FtsZ/GTP systems

To probe whether the increase of scattering observed in the right-angled light-scattering experiments was in fact due to linear FtsZ polymers or some other structures we undertook *LD* experiments as a function of time since *LD* also provides data on any geometry change of sub-units of the FtsZ during the polymerization processes.

**Figure 4:**
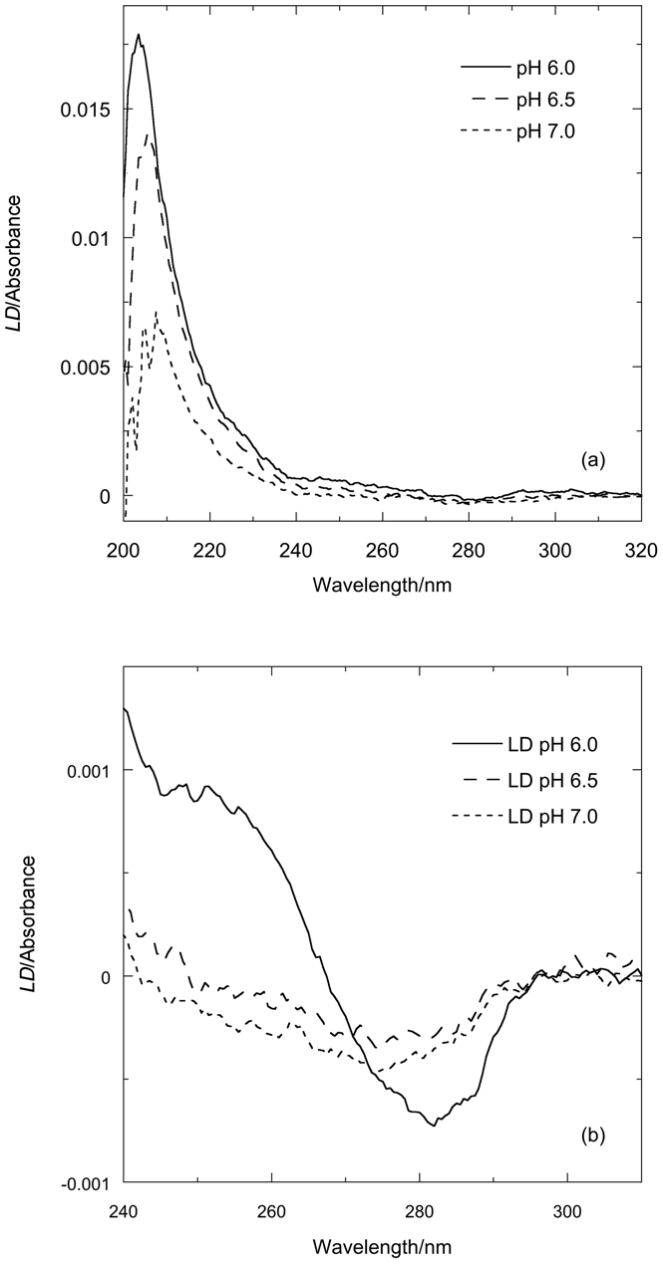
*LD* spectra of FtsZ as a function of pH. *LD* spectra of FtsZ (11 µM) polymers in (MES buffer (50 mM MES-KOH buffer), KCl (50 mM) and MgCl_2_ (10 mM MgCl_2_) 1 minute after addition of GTP (0.2 mM) (a) far UV pH 6.0, 6.5, and 7.0 and (b) near-UV pH 6.0, 6.5, and 7.0 showing the change in sign in one of the guanine transitions (long axis) at ∼250 nm from negative to positive at the lower pH.

The *LD* of FtsZ before the addition of GTP is zero; after the addition of GTP wavelength *LD* scans of FtsZ polymers show (Figure 4a) a positive sign in the 220 nm region. As β-sheets have little or no *LD* at 220 nm, this signal can be ascribed to the *n*→*π* ^*^ transition of the α-helices in FtsZ. This indicates that on average the *n*→*π* ^*^ transition moments, which are oriented perpendicular to α-helix axes, are more parallel than perpendicular to the fibre axis leading us to conclude that the helices within the protein are on average oriented more perpendicular than parallel to the orientation axis [25]. In the near UV region (Figure 4b), the spectrum is dominated by transitions of the aromatic constituents, in particular the tyrosines and the guanine base of the GTP (*E. coli* FtsZ has no tryptophans). The signal at 250 nm in the spectra corresponds to the in-plane long axis *π*→*π* ^*^ transition of the guanine base on the GTP and the 280 nm region to an approximately short axis polarized transition [26]. The negative signs of both transitions at the higher pHs indicates that the guanine lies more perpendicular than parallel to the fibre axis as noted previously [27]. The long axis guanine transition at ∼250 nm, however, changes from negative to positive as the pH decreases indicating a changing tilt of this transition moment, which in turn indicates a reorientation of the guanine as previously noticed in the presence of Ca^2+^-induced bundling of protofilaments [27]. Since this reorientation corresponds to an increase in magnitude of the backbone signal we can conclude that the polymers formed at acidic pH are more oriented (*i.e.* longer and/or more rigid).

### Linear dichroism of FtsZ/GTP as a function of time

**Figure 5:**
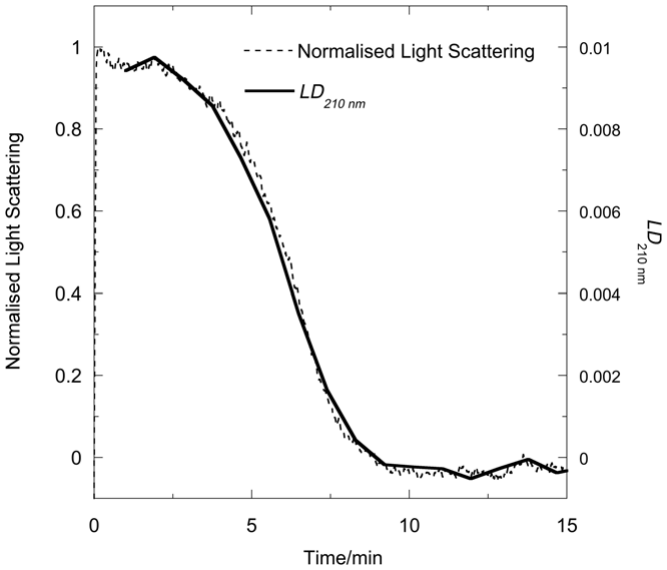
Polymerisation and depolymerisation of FtsZ. FtsZ (11 µM) in polymerisation buffer (50 mM MES buffer pH 6.5, 10 mM MgCl_2_ and 50 mM KCl) monitored by light scattering and *LD*_210_. Freshly thawed GTP (0.2 mM) was added at 0 minutes.

Before using *LD* to follow the kinetics of polymerization or depolymerization we performed identical *LD* and light scattering experiments (Figure 5, pH = 6.5) to facilitate the interpretation of the information provided by *LD*. The *LD*_210_ nm of Figure 5 is the *LD* interval scan signal at 210 nm (which corresponds to the backbone of the polymer) plotted as a function of time. As is shown in Figure 5, the two traces, when plotted so their maximum and minimum signals align, almost overlay enabling us to conclude that the shear forces do not affect the kinetics of depolymerization.

There are subtle difference to which we return below, but in general the *LD* data indicate the early large increase in the *LD* corresponds to the formation of long and/or fairly rigid polymers essentially in the deadtime of the *LD* experiment. The plateau region from 0–4 minutes is where the GTP is turned over and the fibre population remains the same. The final region is a sigmoidal decrease in the *LD* as the polymers dissociate back to monomers (once GTP is depleted).

**Figure 6:**
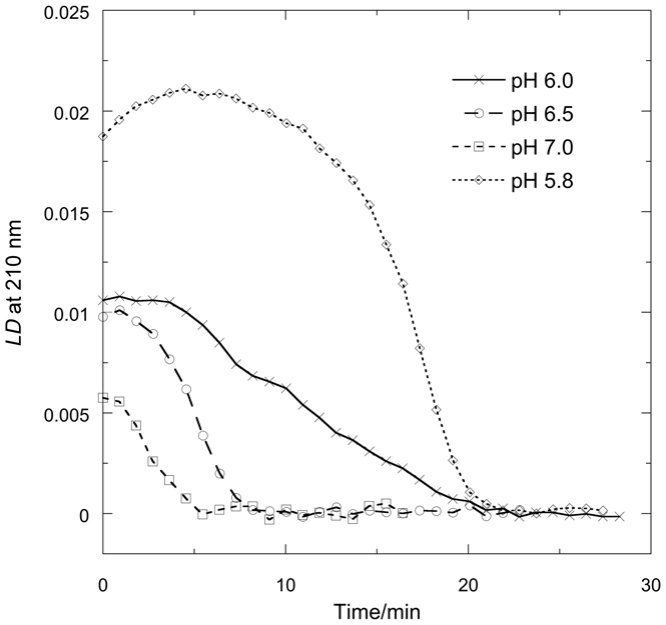
*LD*_210 nm_ of FtsZ. FtsZ (11 µM) in MES buffer (50 mM pH as indicated in figure), KCl (50 mM) and MgCl_2_ (10 mM) with addition GTP (0.2 mM).

Figure 6 shows the time and pH dependence of the *LD* at 210 nm. For each pH, the initial large increase in the *LD* (and light scattering as shown by the high tension voltage trace, data not shown) occurs in the dead-time of the experiment. pH 5.8 data are also shown as the polymerization process is slower so initial increase to maximum signal, similar to the pH 6.0 in the light scattering, is observed in this experiment. The plateau region is longer at low pH indicating a much slower turn-over of the GTP (respectively 5, 2.5 and 2 minute (including 1 minute deadtime) for pH 6, 6.5 and 7), though this region is somewhat shorter than that indicated by the light scattering. The dissociation phase is also slower for lower pH's. Compared with the light sattering, the depolymerization measured by *LD* appears to take somewhat longer.

### Phosphate release

#### Calibration curve for inorganic phosphate

**Figure 7:**
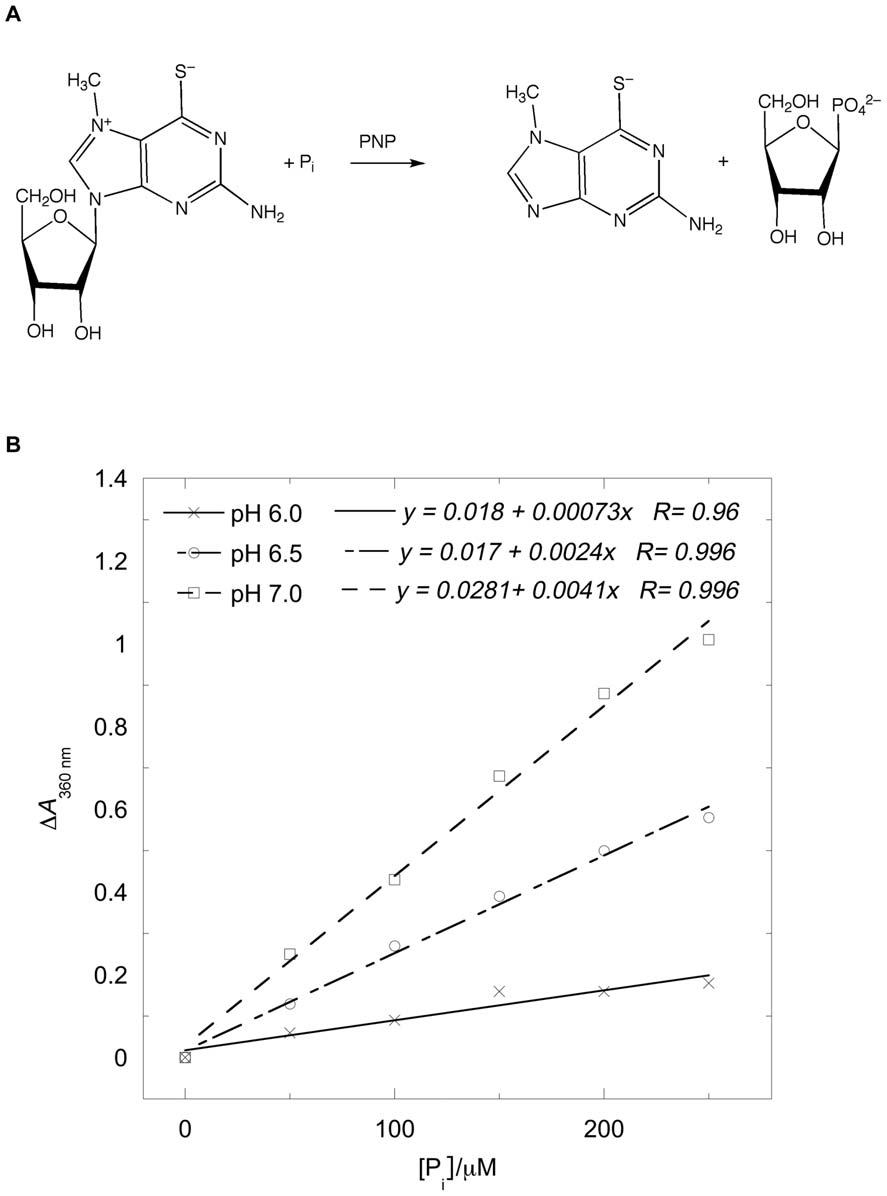
Change in absorbance at 360 nm plotted as a function of the [P_i_] as a function of pH together with the reaction scheme.

We used the phosphate standard provided in the Enzchek Phosphate Assay (Invitrogen) kit (KH_2_PO_4_ (50 mM) with sodium azide (2 mM)) as a source of P_i_ to generate a calibration curve for the inorganic phosphate assay. According to the manufacturer, the linear range of the assay is between 2 and 150 µM and the reaction can be performed over a pH range from 6.0 to 8.5. We required data for 200 µM GTP, so we used a concentration of 0.4 mM MESG instead of the 0.2 mM MESG recommended for the standard reaction and a 5 mm pathlength (instead of 1 cm). Figure 7 shows the change in absorbance at 360 nm plotted as a function of the P_i_ concentration. For the 3 pHs studied, the linear response of the system has been increased to 250 µM P_i_ and accurate data could be obtained even a pH 5.5 (not shown).

#### P_i_ released by FtsZ/GTP mixtures

**Figure 8:**
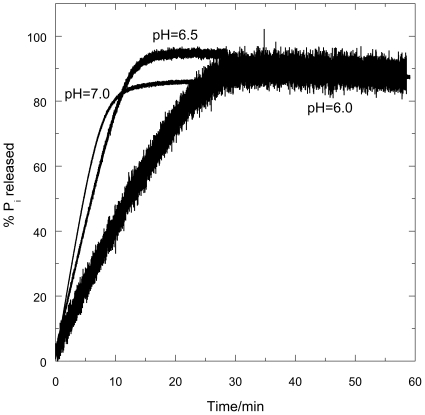
P_i_ released from the enzymatic conversion of GTP to GDP by FtsZ in the presence of MESG as a function of time, *t*. FtsZ (11 µM in MES buffer, pH = 6.0, 6.5 and 7.0); MESG (initial concentration 400 µM). GTP was added to the mixture at *t* = 0. The change in absorbance at 360 nm was measured in a 0.5 cm cuvette and converted to percentage P_i_ release with 100% denoting complete conversion of 0.2 mM GTP according to the calibration curves in Figure 7. The experimental error for the assay is in the range of 5%.

The hydrolysis of GTP to GDP and P_i_ by FtsZ was followed as a function of time using the modified phosphate assay. Absorbance was monitored during the 10 min pre-incubation time to check for possible P_i_ contamination: there was none. In Figure 8, *t* = 0 is the point where GTP was added and the vertical axis is the percentage of P_i_ released at any given time where 100% corresponds to complete hydrolysis of GTP to GDP.

Several observations follow from Figure 8:

- At all pHs studied, P_i_ was released upon addition of GTP.
- The signal to noise ratio observed for pH 6.0 is lower than that observed at pH 7.0 simply due to the fact that the ΔA_360_ at pH 6.0 is very small compared to the ΔA_360_ at pH 7.0 due to pH-dependence of the absorbances.
- The P_i_ release is faster at higher pH: the reaction took 30 minutes to complete at pH 6.0; 15 minutes at pH 6.5; and 10 minutes at pH 7.0—all of which are longer times than both the light scattering and *LD* suggest.
- The endpoint was 90% of the theoretical value at pH 6.0; 96% at pH 6.5; and 87% at pH 7.0.

### GDP/GTP competition

**Figure 9:**
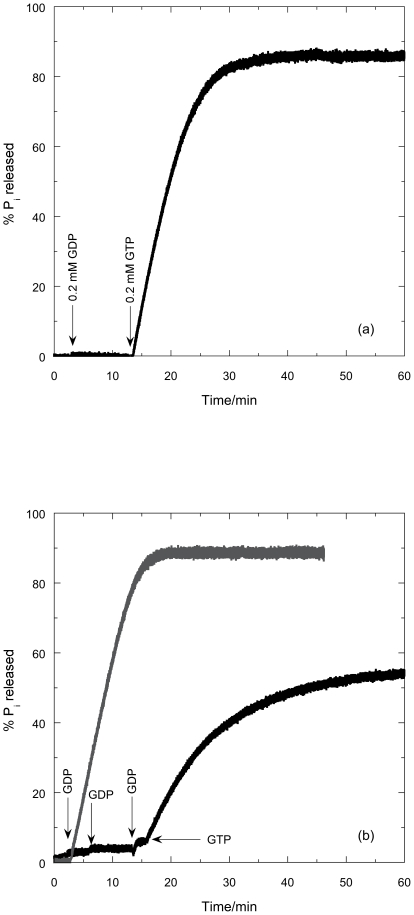
P_i_ release when GTP and GDP are sequentially added to FtsZ. (a) GDP (0.2 mM) and GTP (0.2 mM) and (b) GDP (0.2 mM), GDP (0.2 mM), GDP (0.67 mM), GTP (0.2 mM) are sequentially added as indicated to FtsZ (11 µM in MES buffer, pH 6.5) in the presence of MESG (initial concentration 400 µM) as a function of time. Figure (a) curve is overlaid in (b) in grey with an axis offset to align the first GDP addition. The change in absorbance at 360 nm was measured in a 0.5 cm cuvette and converted to percentage P_i_ release with 100% denoting complete conversion of 0.2 mM GTP according to the calibration curves in Figure 6. The experimental error for the assay is in the range of 5%.

If GDP (final concentration 0.2 mM) is added (at 3 minutes) instead of GTP, no P_i_ release was observed (first phase of Figure 9a). When GTP (0.2 mM) was added at 14 minutes the percentage of P_i_ released is, within experimental error, the same as the case where no GDP was initially present in the sample. When, however, the total GDP added is over 4 times as much as the GTP (Figure 9b), so 80 times as much as the FtsZ, the P_i_ release is much slower than without the excess GDP: after 44 minutes, only 55% of the theoretical maximum P_i_ has been released.

### Electron microscopy

**Figure 10:**
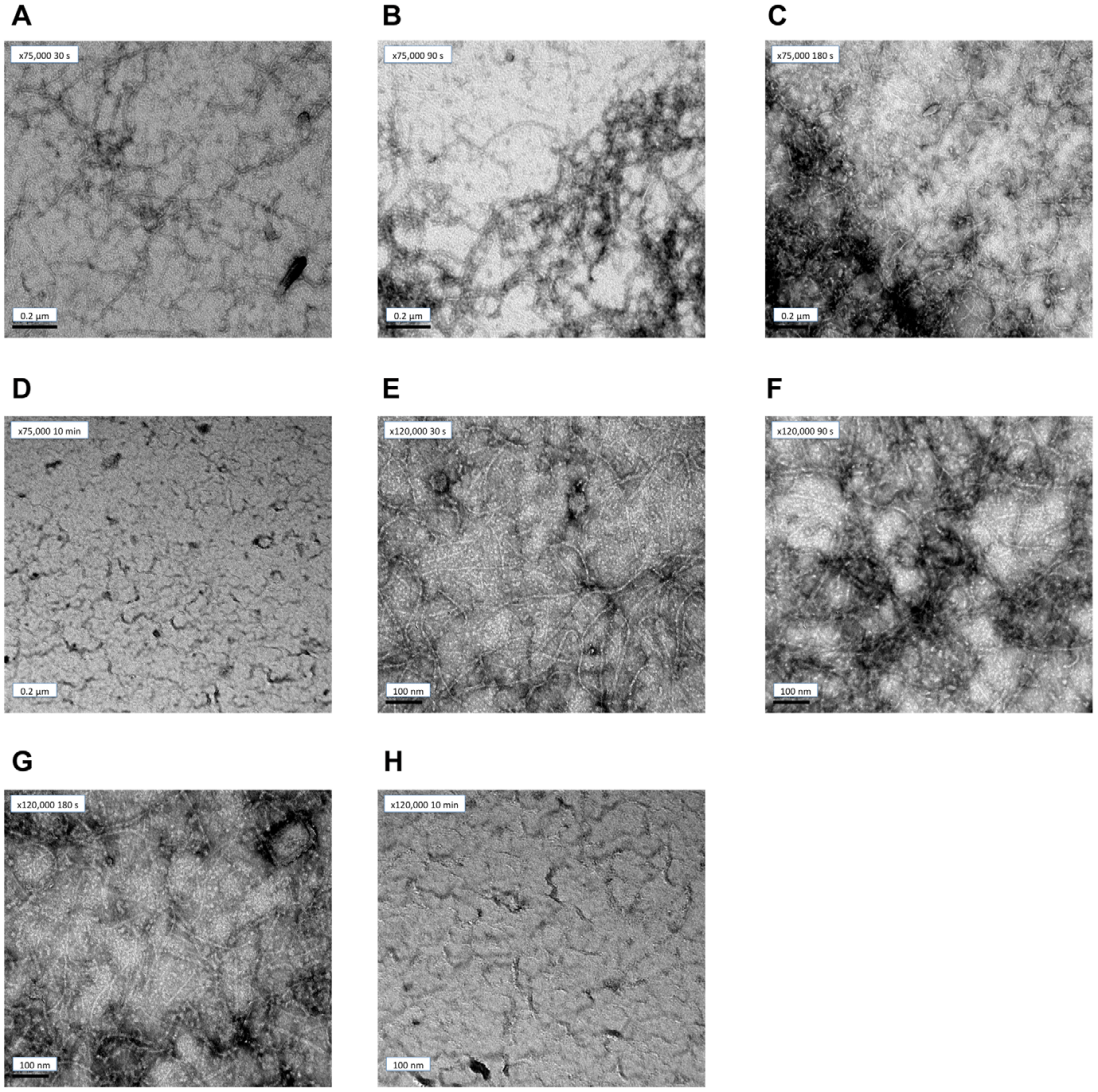
EM images of FtsZ polymerisation at different time points. FtsZ (11 µM) was incubated in the polymerisation buffer (pH = 6.5) for 15 minutes prior to the addition of GTP (0.2 mM). At the indicated times the reaction was stopped and the samples were negatively stained by the addition of 1% uranyl acetate. Scale bar represents 0.2 µm for ×75,000 magnification and 100 nm for ×120,000 magnification.

As shown in Figure 10 at pH = 6.5 a dense network of protofilaments was present 30 seconds (Figure 10a) after the addition of GTP and had reached steady state by 3 mins (Figure 10c). The protofilaments formed under the reactions conditions used in this work were typically 5 nm in diameter and tend to align along their long axis in structures composed from 2 to several protofilaments. By 6 minutes (data not shown), the protofilaments were shorter and less abundant and by 10 minutes the protofilaments were no longer visible (Figure 10d). We may thus conclude that the increase in light scattering observed in Figure 3 is due to the polymerization of FtsZ after GTP addition and the decrease in light scattering attributed to depolymerisation after GTP is depleted.

**Figure 11:**
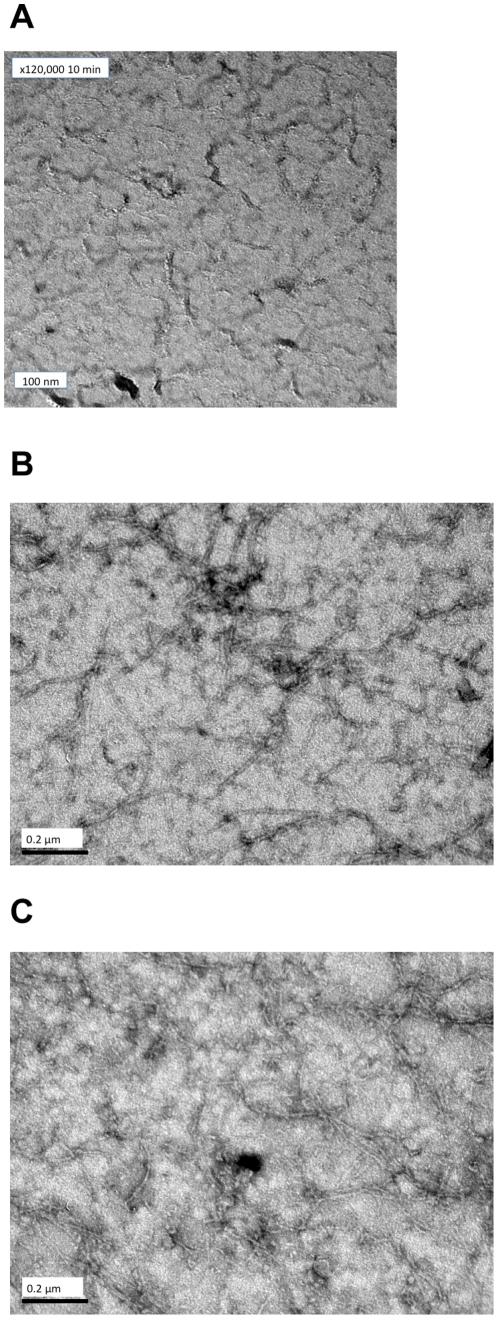
FtsZ (11 µM) incubated in the polymerisation buffer (50 mM MES). (a) pH = 6.0, (b) pH = 6.5, (c) pH = 7, for 15 minutes prior to the addition of GTP (0.2 mM). The reactions were stopped at 4 mins after GTP addition and samples were negatively stained by the addition of 1% uranyl acetate. Selected areas were photographed at ×75,000. Scale bar represents 0.2 µm.

The protofilaments formed at pH 6.0 (Figure 11a ×75,000) consisted of a dense network of protofilaments that were long and highly laterally associated. The polymers formed at pH 6.5 were long and form a dense network of protofilaments (Figure 11b ×75,000) with a small degree of lateral association. At pH 7.0 (Figure 11c ×75,000), the protofilaments were shorter, less abundant and even less laterally associated than those formed at pH 6.5.

## Discussion

FtsZ polymer dynamics are controlled during the cell cycle by a balanced network of positive- and negative-acting factors. An exhaustive understanding of FtsZ dynamics will be very likely to depend on an extension and/or development of the range of techniques currently being used in different laboratories. We have therefore developed methods to enable us to get a more detailed picture of the molecular level behaviour of the process.

The phosphate assay provides an indirect measure of the catalytic activity of FtsZ since polymeric FtsZ is a GTPase (*i.e.* it hydrolyses GTP into GDP and inorganic phosphate, P_i_). The sensitivity of the method has been successfully modified in this work to cover at least the range 2 µM–250 µM P_i_ and the data are reliable over a pH range of 5.5–7.0. This methodology probes the total phosphate release, whether it comes from small or large polymers.

The light scattering and *LD* assays at first sight provide much the same view of the polymerization process: both detect the formation of polymers and give larger signals for bundles of polymers, and do not detect small polymers. However, there are subtle difference between light scattering and *LD* relating to the fact that the light scattering is dependent only on polymer mass whereas *LD* is dependent on the orientability of the polymers: a larger *LD* signal correlates with longer or stiffer fibres. In the case of the FtsZ polymers this means that *LD* increases with the extra stiffness engendered by the bundling process (rather than simply an increase in size which is what the light scattering reflects) whereas any curvature of the polymers, as is thought to occur with GDP-containg fibres and more mildly in some GTP fibres [28], will shorten the polymers so reduce the *LD* but have little effect on the light scattering. The molecular conclusions that can be drawn from our data are summarized below.

### GDP

Addition of GDP did not result in any P_i_ release, confirming that GTP hydrolysis is the source of the P_i_. GDP does, however, inhibit hydrolysis if it is in sufficient excess to block the active site.

### Kinetics

The kinetics of polymerization measured by light scattering and *LD* at pH 6.5 are very similar. The subtle differences illustrated in Figure 5 (and also apparent by a comparison of Figures 3b and 6), however, show that the plateau region is less obvious in the *LD* and is apparently over sooner. The phosphate release data have no plateau region at all: Figure 8 indicates that the P_i_ release at pH 6.5 is steady until the reaction stops well after (∼3 minutes after) the polymers have dissociated according to the other techniques. So the three methods which are at first sight are following the same process, are in fact providing complementary information. The phosphate release requires only the formation of an active hydrolysis site—so detects any size upwards from dimers, which are invisible to the other techniques. The fact that the phosphate release starts immediately the GTP is added means that the polymers always contain some GDP. It has been established that the GDP in the polymers exchanges with GTP available in solution [29]. GDP also causes FtsZ protofilament curvature [30]. Such curvature would reduce the *LD* signal without reducing the light scattering as polymer mass is retained. Arguably there is more curvature in the 3 minute EM pictures than at 30 s which is consistent with the *LD* disassembly phase appearing to start before the light scattering disassembly.

### pH dependence

Mukherjee and Lutkenhaus [11] studied the pH dependence of FtsZ kinetics. They found that FtsZ readily formed polymers at pH = 6.0, 6.5 and 7.2 with the only difference being the abundance of the polymers (more abundant at pH 6.5). The results of this present work show more details about the differences. The EM pictures show that pH 6.5 did indeed produce more polymers; those at pH 6.0 show more bundling and those at pH 7.0 least.

The *LD* shows that the polymers formed at the lowest pH orient better, and have tilted guanines (which we have previously seen to occur when the protofilaments bundle) [8]. The bundled polymers also depolymerize more slowly. Intriguingly, the % completion of phosphate release is greatest at pH 6.5 (where it is mainly a population of long protofilaments). The phosphate release assay measures released P_i_ so the differences in its rates are due to a combination of rate of hydrolysis and the release of P_i_ into solution. At pH 6.5 and 7.0, the polymers are basically single protofilaments with little lateral association as shown by electron microscopy (Figure 11). The slower kinetics at pH 6.5 (compared with pH 7) suggest this is an effect of the pH on the actual hydrolysis. The greater population of polymers at pH 6.5 is in accord with the total higher % P_i_ release. The slower release at pH 6.0 could either be due to slower hydrolysis in the bundles of fibres or to slower release of P_i_ after hydrolysis (or in this case probably both). GDP and P_i_ held in an active site in a bundle will not readily be released into solution so exchange with a new GTP cannot occur. The lower total % P_i_ release at pH 6.0 reflects the lower effective active site concentration due to the bundling of protofilaments.

### Significance of pH dependence

Perhaps the most important result from this work for our attempts to understand FtsZ polymerization is that lowering the pH from neutral to 6.5 does not change the nature of the FtsZ polymers in solution—it simply facilitates the polymerization so the fibres present are longer and more abundant. Conversely, lowering the pH to 6.0 (still well-above the PI of 4.59) has much the same effect as introducing divalent cations or the FtsZ-associating protein YgfE (a putative ZapA orthologue in *E. coli*)—it stablizes the associations of protofilaments. The lower hydrolysis at lower pH is not merely due to any chance in the GTP as proposed in [13]. This pH dependence may be important in maintaining the integrity of the Z-ring and it may play an important role in the structure of the macromolecular ring, however, the biological relevance is not clear.

## References

[1] Adams DW, Errington J (2009). Bacterial cell division: assembly, maintenance and disassembly of the Z ring.. Nat Rev Microbiol.

[2] Errington J, Daniel RA, Scheffers DJ (2003). Cytokinesis in bacteria.. Microbiol Mol Biol Rev.

[3] Bramhill D, Thompson CM (1994). GTP-dependent polymerisation of *Escherichia coli* FTsZ protein to form tubules.. Proc Natl Acad Sci.

[4] Mukherjee A, Lutkenhaus J (1994). Guanine nucleotide-dependent assembly of FtsZ into filaments.. Journal of Bacteriology.

[5] Stricker J, Maddox P, Salmon ED, Erickson HP (2002). Rapid assembly dynamics of the *Escherichia coli* FtsZ-ring demonstrated by fluorescence recovery after photobleaching.. Proc Natl Acad Sci.

[6] Haydon DJ, Stokes NR, Ure R, Galbraith G, Bennett JM (2008). An inhibitor of FtsZ with potent and selective anti-staphylococcal activity.. Science.

[7] Chen Y, Erickson HP (2009). FtsZ Filament Dynamics at Steady State: Subunit Exchange with and without Nucleotide Hydrolysis.. Biochemistry.

[8] Marrington R, Small E, Rodger A, Dafforn TR, Addinall S (2004). FtsZ fibre bundling is triggered by a calcium-induced conformational change in bound GTP.. J Biol Chem.

[9] Small E, Marrington R, Rodger A, Scott DJ, K. S (2007). FtsZ polymer-bundling by the Escherichia coli ZapA orthologue, YgfE involves a conformational change in bound GTP.. J Mol Biol.

[10] Wilks JC, Slonczewski JL (2007). pH of the cytoplasm and periplasm of Escherichia coli: rapid measurement by green fluorescent protein fluorimetry.. J Bacteriol.

[11] Mukherjee A, Lutkenhaus J (1998). Dynamic assembly of FtsZ regulated by GTP hydrolysis.. EMBO Journal.

[12] Scheffers D-J (2008). The effect of MinC on FtsZ polymerization is pH dependent and can be counteracted by ZapA.. FEBS Lett.

[13] Mendieta J, Rico AI, López-Viñas E, Vicente M, Mingorance J (2009). Structural and Functional Model for Ionic (K+/Na+) and pH Dependence of GTPase Activity and Polymerization of FtsZ, the Prokaryotic Ortholog of Tubulin.. J Mol Biol.

[14] Bradford M (1976). A Rapid and Sensitive Method for the Quantitation of Microgram Quantities of Protein Utilizing the Principle of Protein-Dye Binding.. Anal Biochem.

[15] Mukherjee A, Lutkenhaus J (1998). Purification, Assembly and Localization of FtsZ.. Methods on Enzymology.

[16] Small E, Addinall SG (2003). Dynamic FtsZ polymerization is sensitive to the GTP to GDP ratio and can be maintained at steady state using a GTP-regeneration system.. Microbiology.

[17] Mukherjee AaL, J. (1999). Analysis of FtsZ assembly by light scattering and determination of the role of divalent metal cations.. Journal of Bacteriology.

[18] Marrington R, Dafforn TR, Halsall DJ, Macdonald JI, Hicks M (2005). Validation of new microvolume Couette linear dichroism cells.. Analyst.

[19] Marrington R (2004). Polarised spectroscopy of biomacromolecules.

[20] Nordh J, Deinum J, Norden B (1986). Flow orientation of brain microtubules studied by linear dichroism.. European Biophysical Journal.

[21] Marrington R, Seymour M, Rodger A (2006). A new method for fibrous protein analysis illustrated by application to tubulin microtubule polymerisation and depolymerisation.. Chirality.

[22] Webb MR, Biochemistry (1992). A continuous spectrophotometric assay for inorganic phosphate and for measuring phosphate release kinetics in biological systems.. Proc Natl Acad Sci.

[23] Johnson WC (1999). Analyzing protein circular dichroism spectra for accurate secondary structures.. Proteins Struct Funct Genet.

[24] Johnson WC (1988). Secondary structure of proteins through circular dichroism spectroscopy.. Ann Rev Biophys Biophys Chem.

[25] Rodger A, Rajendra J, Marrington R, Ardhamar M, Norden B (2002). Membranes and proteins.. Phys Chem Chem Phys.

[26] Nordén B, Rodger A, Dafforn TR (2010). Linear dichroism and circular dichroism: a textbook on polarized spectroscopy.

[27] Marrington R, Addinall S, Dafforn TR, Small E, Rodger A (2004). Calcium induced conformational change in GTP bound to FtsZ: A trigger for fibre bundling.. In Progress.

[28] Erickson HP (2009). Modeling the physics of FtsZ assembly and force generation.. Proc Nat Acad Sci.

[29] Mingorance J, Rueda S, Gomez-Puertas P, Valencia A, Vicente M (2001). Escherichia coli FtsZ polymers contain mostly GTP and have a high nucleotide turnover.. Molecular Microbiology.

[30] Lu C, Reedy M, Erickson HP (2000). Straight and curved conformations of FtsZ are regulated by GTP hydrolysis.. J Bacteriol.

